# Reduction of Dopamine Level Enhances the Attractiveness of Male *Drosophila* to Other Males

**DOI:** 10.1371/journal.pone.0004574

**Published:** 2009-02-24

**Authors:** Tong Liu, Laurence Dartevelle, Chunyan Yuan, Hongping Wei, Ying Wang, Jean-François Ferveur, Aike Guo

**Affiliations:** 1 Institute of Neuroscience, State Key Laboratory for Neuroscience, Shanghai Institutes for Biological Sciences, Chinese Academy of Sciences, Shanghai, China; 2 Graduate school of Chinese Academy of Sciences, Beijing, China; 3 State Key Laboratory of Brain and Cognitive Science, Institute of Biophysics, CAS, Beijing, China; 4 CNRS-UMRS 5548, Universite de Bourgogne, Dijon, France; Pennsylvania State University, United States of America

## Abstract

Dopamine is an important neuromodulator in animals and its roles in mammalian sexual behavior are extensively studied. *Drosophila* as a useful model system is widely used in many fields of biological studies. It has been reported that dopamine reduction can affect female receptivity in *Drosophila* and leave male-female courtship behavior unaffected. Here, we used genetic and pharmacological approaches to decrease the dopamine level in dopaminergic cells in *Drosophila*, and investigated the consequence of this manipulation on male homosexual courtship behavior. We find that reduction of dopamine level can induce *Drosophila* male-male courtship behavior, and that this behavior is mainly due to the increased male attractiveness or decreased aversiveness towards other males, but not to their enhanced propensity to court other males. Chemical signal input probably plays a crucial role in the male-male courtship induced by the courtees with reduction of dopamine. Our finding provides insight into the relationship between the dopamine reduction and male-male courtship behavior, and hints dopamine level is important for controlling *Drosophila* courtship behavior.

## Introduction

Courtship behavior is fundamental to the propagation of animal species and their adaptation to circumstances. Animals use multiple sensory cues to recognize conspecifics and to choose a potentially suitable mate for reproduction [1], [2]. Male courtship in *Drosophila* is a complex behavior which consists of a stereotypic set of behaviors, including orientation towards a female, taping with its forelegs, following the female, singing courtship song, licking the female's genital, attempting copulation, and finally copulation [3], [4]. *Drosophila* courtship behavior depends on visual, acoustic and chemosensory signals exchanged by the two partner flies [3], [4], [5], some of which can stimulate male or female courtship while others can inhibit it [6]. Thus, abnormal courtship behavior can be induced by either the change of courter's sensory perception or the change of courtee's attractiveness and receptivity.

Dopamine (DA) is a very important neuromodulator in animals. Its roles in mammal sexual behavior has been extensively studied [7], [8]. In insects, DA is used as a neurotransmitter in the central nervous system and as a cuticle hardening and pigmenting factor in the epidermis [9], [10]. Tyrosine hydroxylase (TH) is the rate-limiting enzyme for DA synthesis. Studies demonstrate that DA elevation can enhance male-male courtship behavior in *Drosophila* [11] and that DA reduction affects specific aspects of courtship behavior: female sexual receptivity to males and male courtship conditioning towards immature males [12], [13], but did not change male courtship towards females [12]. Since the effect of DA reduction on male-male courtship has not been reported before, we used genetic and pharmacological tools to investigate the relationship between DA reduction and male-male courtship. We found that DA reduction could induce male-male courtship by enhancing the attractiveness of courtees.

## Results

**Figure 1:**
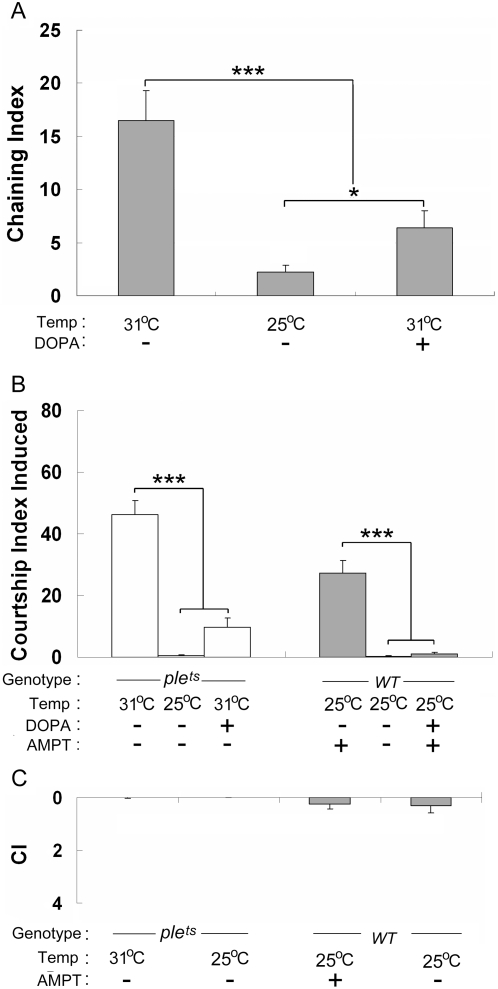
Dopamine reduction and enhanced male homosexual attractiveness. A, Chaining index (ChI) for *ple^ts1^* males reared at 25°C or at 31°C either in plain medium (−) or in medium supplemented with L-DOPA (+). N = 48–59. B, The amount of CI induced either by *ple^ts1^* males (light bars) or by wild-type males (filled bars) reared either on plain food at 25°C or at 31°C, eventually with L-DOPA-enriched food (+). Wild-type males were always reared at 25°C on plain food (−), or on food containing AMPT eventually supplemented with L-DOPA (+). N = 30–49, except for *ple^ts1^* males reared at 25°C (17). C, Inverted bars represent the courtship index (CI) directed to wild-type males by mutant *ple^ts1^* or wild-type males (WT) males raised either at 25°C or at 31°C, on plain food (−) or on AMPT-enriched food (+). N = 32–47 except for *ple^ts1^* males reared at 25°C (17). Each histogram represents the mean behavioral index with its s.e.m. *** indicates p<0.001; and * p<0.05 (For two-group comparison, Mann-Whitney Rank Sum test was used; For more than 2-groups comparison, ANOVA on Rank test was used).

The flies we used carried a temperature-sensitive allele of the *pale* gene (*ple^ts1^*; 25°C is the permissive temperature, 31°C is the restrictive temperature) [14] which encodes TH that is expressed in both larval and adult nervous systems and also in the larval epidermis [15]. To test the efficiency of *ple^ts1^* mutation, we measured the DA content in the *ple^ts1^* males raised at 25°C (18.7±2 ng/mg mass, s.e.m. N = 5) and at 31°C (8.6±1 ng/mg mass, s.e.m. N = 6) respectively. The data showed that the *ple^ts1^* males raised at 31°C indeed had significant lower level of DA as compared with sibling males raised at 25°C (p = 0.004, Mann-Whitney Rank Sum test), which indicates *ple^ts1^* mutation can efficiently decrease DA level at the restrictive temperature. Males homozygous for the *ple^ts1^* allele showed markedly different chaining behavior (measured by the chaining index, ChI; Fig. 1A) according to the temperature at which they were raised during adulthood. Males continuously raised at 25°C showed rare male-male interaction while sibling males raised at 31°C showed a markedly increased ChI (Fig. 1A). This behavioral variation is likely related to the DA reduction induced by *ple^ts1^* mutation since this defect could be rescued by the addition of DA precursor (L-DOPA) in the food. Indeed, males raised at 31°C and fed during adulthood with L-DOPA showed a much lower ChI than sibling males raised at 31°C on unsupplemented food.

The chaining behavior observed between manipulated males could result from the reciprocal alteration of either the courter, the courtee, or both partners. To clearly assess the behavior of each partner, we measured the courtship index (CI) directed or received by individual mutant *ple^ts1^* males when paired with single wild-type males. When mutant males were used as targets, the CI that they received from wild-type males varied according to their treatment: *ple^ts1^* males raised at 31°C on standard food induced a high CI (Fig. 1B; Movie S1), but when raised at 25°C on standard food, they induced no courtship. Males raised at 31°C on L-DOPA-supplemented food received a low CI from wild-type males. The wild-type males raised at 31°C also showed an obvious attractiveness for the wild-type males (CI = 12.94±2.4 s.e.m., N = 68). The male-male courtship behavior induced by *ple^ts1^* males raised on L-DOPA-supplemented food at 31°C was not significantly different as compared with the wild-type males raised on unsupplemented food at 31°C (CI*_ple_^ts1^* = 9.78±2.9 s.e.m.; CI_wild-type_ = 12.94±2.4 s.e.m.; p = 0.14, Mann-Whitney Rank Sum test), but was significantly higher than the sibling raised on unsupplemented food at 25°C (Fig. 1A,B), which indicates that temperature also has an effect on the male-male courtship behavior.

In the reciprocal interaction, *ple^ts1^* males, raised at either temperature, directed a very low CI to wild-type target males (Fig. 1C). Since temperature showed an obvious effect on this kind of male-male courtship behavior, we used a complementary strategy to rule out the effect of temperature on the attractiveness [16] and to confirm the implication of DA reduction in male homosexual attractiveness (the male tendency to stimulate the courtship of other males, as opposed to the male propensity to court other males). Wild-type males were raised at 25°C and fed with AMPT, a drug that tends to reduce DA synthesis. Wild-type males fed with AMPT elicited a strong CI of control males whereas no reciprocal interaction was noted (Fig. 1B,C). This effect is similar to that observed in *ple^ts1^* males raised at 31°C. Wild-type males simultaneously fed with AMPT and L-DOPA elicited no CI of untreated sibling males (Fig. 1B). Taken together, these genetic and pharmacological experiments indicate that DA reduction enhanced male attractiveness — but did not change their courtship — to other males.

**Figure 2:**
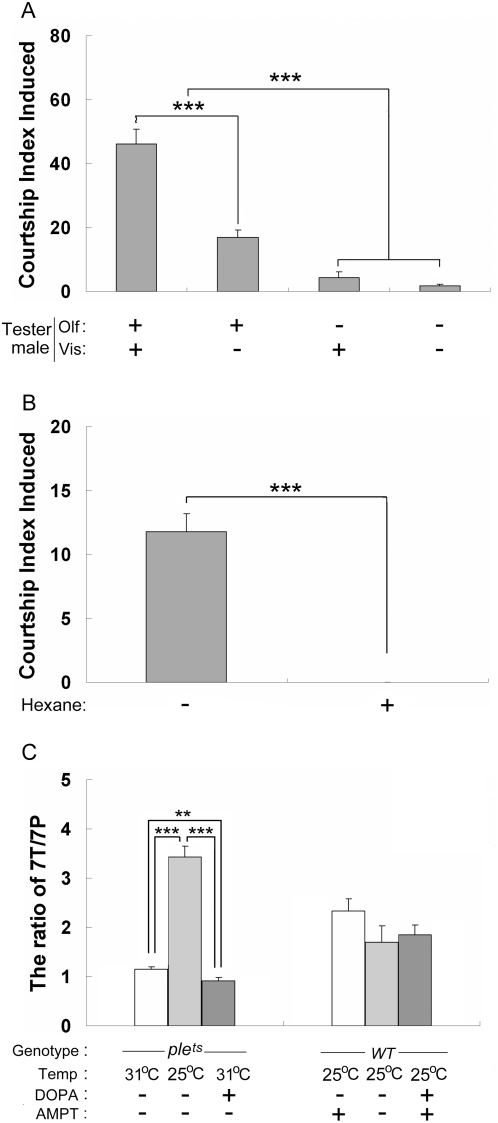
The effect of sensory input deprivation on the male-male courtship and the measurement of cuticular hydrocarbons. A, The CI of manipulated wild type males with normal or altered sensory systems to intact *ple^ts1^* males reared at 31°C. The manipulation of olfactory/auditory (Olf) and visual (Vis) stimuli is indicated at the bottom: presence (+), absence (−). N = 32, 80, 22 and 28 respectively. B, The CI of intact wild type males to decapitated *ple^ts1^* males (−) and hexane-rinsed *ple^ts1^* males (+) reared at 31°C respectively. N = 63 and 26 respectively. C, The ratio of two main male cuticular pheromones 7-T and 7-P for the male treatment indicated. N = 13–15. Each histogram represents the mean behavioral index or 7T/7P ratio with its s.e.m. *** indicates p<0.001 (In A and B, for two-group comparison, Mann-Whitney Rank Sum test was used, and for more than 2-groups comparison, ANOVA on Rank test was used; in C, Student's t-test was used).

*Drosophila* male courtship and mate discrimination depend mostly on the perception of visual, pheromonal and acoustic signals exchanged by partner flies [6]. To assess the involvement of these signals in the attractiveness shown by males with reduced DA level, several experiments were designed to eliminate the effect of each type of signal. We measured the CI induced by males under different sensory deprived condition (Fig. 2A). To eliminate the visual signals, wild-type males (used as courters) were tested with intact *ple^ts1^* mutant males raised at 31°C under red light where they are virtually blind [17]. They were also surgically ablated both their antennae and maxillary palps (NAM) to reduce their perception of pheromones [17] and of acoustic signals [18]. In another experiment, the *ple^ts1^* mutant males raised at 31°C were washed with hexane to rinse out the pheromones to remove the effects of these olfactory and gustatory inputs (Fig. 2B). Wild-type males either olfactory/auditory- or visually-deprived showed significantly reduced CI to intact *ple^ts1^* mutant males raised at 31°C (Fig. 2A). The deprivation of olfactory/auditory inputs had (i) a more dramatic effect on CI than the visual input deprivation, and (ii) the same effect as the two deprivations simultaneously applied (Fig. 2A). The fact that *ple^ts1^* males washed with hexane completely lost their attractiveness to wild-type males (Fig. 2B) indicates that cuticular chemicals are the critical sensory signals used by male flies with decreased DA level to stimulate the courtship of wild-type males. These signals could either increase male attractiveness or reduce their aversiveness to other males.

To study the identity of these chemical signals, we measured the cuticular pheromones in the diversely manipulated *ple^ts1^* and wild-type males. It has been known that 7-tricosene (7-T; inhibiting male-male courtship) and 7-pentacosene (7-P; stimulating male-male courtship) are the two main pheromones of male fly [19], and the reduced ratio of 7T/7P might enhance the attractiveness of males. A significantly decreased ratio of 7T/7P was observed in the *ple^ts1^* males raised at 31°C as compared with the sibling males at 25°C (Fig. 2C). Since it has been reported that temperature change can affect the expression pattern of pheromones [20], the cuticular pheromones of wild-type male raised at different temperatures were measured and the data showed the ratio of 7T/7P in wild-type males raised at 31°C was also much lower than the sibling males raised at 25°C (7T/7P_25°C_ = 1.70±0.3 s.e.m.; 7T/7P_31°C_ = 0.65±0.06 s.e.m.; p = 0.01, Student's t-test). The ratio of 7T/7P in *ple^ts1^* males raised at 31°C was significantly higher than that of wild-type males raised at the same temperature (p<0.001, Student's t-test), yet they showed much more attractiveness to other males as compared with wild-type males (p<0.001, Mann-Whitney Rank Sum test). This suggested that the decreased ratio of 7T/7P in *ple^ts1^* males raised at 31°C was probably not the reason for their attractiveness. Further experiments proved that the lower ratio of 7T/7P was not responsible for the male attractiveness induced by DA reduction, because that: (i) the *ple^ts1^* males raised on L-DOPA-supplemented food at 31°C showed significantly lower ratio of 7T/7P than that of *ple^ts1^* males raised on unsupplemented food 31°C, but their attractiveness was obviously reduced (Fig. 2C; p = 0.01, Student's t-test); (ii) The ratio of 7T/7P in the wild-type males with low level of DA produced by pharmacological method was not statistically different as compared with the sibling males raised on unsupplemented food or on L-DOPA&AMPT-supplemented food at 25°C (Fig. 2C; Student's t-test), but these male flies still showed obvious attractiveness. Particularly, no correlation was found with the ratio of the two principal cuticular pheromones: 7-T and 7-P, nor with either the percentage or the absolute amount of any other minor secondary compounds between 23 carbons and 29 carbons detected on their cuticle with a GC-MS device (not shown). Therefore, our data showed no obvious causal relationship between the intensity of their homosexual attractiveness produced by DA reduction and the variation of their pheromones we tested.

## Discussion

Our study reveals that reduced DA level in males highly increased their attractiveness to wild-type males, but had no effect on their propensity to court other males, and the enhanced male attractiveness was mainly attributed to the change of chemical signals. Although we did not find any candidate compound responsible for the male attractiveness, which was consistent with the result of Marican et al. [21], we suspect that other yet unknown pheromonal compounds, whose level depends upon the DA level, may regulate male attractiveness to other males. The male pigmentation has been proposed to be a sex- and species specific signal for courtship and mating [22], [23]. The pigmentation of bodies was lighter in *ple^ts1^* males raised at the restrictive temperature (31°C) than in males raised at the permissive temperature (25°C; data not shown) [10], [14], suggesting that visual cues might be also involved in the increased attractiveness of *ple^ts1^* mutant males. Furthermore, the decrease of rejection efforts led by reduction of DA level is another possibility for the male-male courtship induced by males with low level of DA.

Although the male attractiveness induced by DA reduction did not depend on the high temperature, which was proved by our pharmacological assay, the data showed that temperature itself could also enhance male attractiveness for other males (CI_wild-type31°C_ = 12.94±2.4 s.e.m.; CI_wild-type25°C_ = 3.80±2.2 s.e.m.; p<0.001, Mann-Whitney Rank Sum test). Then, is the male attractiveness induced by high temperature related to low level of DA? To test this question, we measured the DA content of wild-type males raised at 31°C (19.0±2 ng/mg mass, s.e.m. N = 6) and 25°C (16.8±3 ng/mg mass, s.e.m. N = 6) respectively. Our data showed that there was no significant difference between the DA level of wild-type males raised at 31°C and at 25°C, which indicates that the male attractiveness induced by high temperature is unrelated to the reduction of DA level.

Interestingly, increased DA levels in males can also induce male-male courtship, which is due to the change of the sexual propensity of courters but not the change of the attractiveness of courtees [11]. Either high level or low level of DA can induce male-male courtship behavior, while the mechanism is different. Variations of DA level affect different yet complementary aspects of courtship behavior in *Drosophila* males, which indicates that DA system plays an important role in regulating *Drosophila* male courtship behavior.

Our study provides new insight on the regulation of male attractiveness. The next goal will be to precisely identify the cuticular pheromones affected in the DA-depleted male flies. One challenge will be to understand the biological basis of the reciprocal effect induced by DA variation (increase/decrease) on the modulation of sensory communication and whether this novel finding can be generalized to other animals.

## Materials and Methods

### Fly culture and surgery

The wild-type strain used was *Canton S*. The mutant strains used was *pale^ts1^* (*ple^ts1^*, a temperature-sensitive TH allele) [14]. Flies were collected within 4 hours after eclosion under light CO_2_ anesthesia and reared on unyeasted standard medium (Bloomington) eventually supplemented with different drugs. Flies were reared either at 25°C or 31°C, with 60% relative humidity, and a 12hr∶12hr light-dark photoperiod. Male used in behavioral assays were aged for 6∼8 days when raised at 25°C and 5 days when raised at 31°C after eclosion respectively. The males' antennae&maxillary palps were ablated (NAM) when the flies were one-day-old and the experiments were conducted seven days later. For drug treatment: (*i*) To reduce DA level in wild-type males: 5 mg/ml alpha-Methyl-DL-tyrosine methyl ester hydrochloride (AMPT, Sigma) was used; (*ii*) To increase DA level in *ple^ts1^* males raised at 31°C and in wild-type males raised on AMPT-containing food at 25°C: 3 mg/ml L-DOPA (Sigma) was used. For all drug treatments, flies were collected less than 4 hours after eclosion and raised on drug-containing food, until the test. The *ple^ts1^* males were raised at 25°C before adult eclosion. After eclosion, they were either kept at 25°C and tested when 8 day-old, or raised at 31°C and tested when 5 day-old.

### Behavior

***Paired courtship assay*** was performed in a courtship chamber (12 mm×18 mm) with a pair of flies. The courtship index (CI) is the percentage of time in a 5-min observation period that the tester male spent courting the target fly (this includes tapping, following, wing vibration, attempted copulation) [24]. Since both flies had the same appearance, they were distinguished from each other by a color dot painted on the thorax of the target flies, a few minutes before the experiment. Tester males were reared individually whereas target flies were reared in groups of 40∼50.

***Chaining assay*** involved ten flies introduced in a 95 mm×23 mm tube with fresh food [24]. A chain was defined as a group of at least three males exhibiting courtship behavior [25]. Chaining index (ChI) is the percentage of time that groups of males spent courting during the 10-min observation period. Males were reared in groups of 60∼70.

#### Quantitative determination of dopamine [26]

Adult male flies were collected and quick-frozen in liquid nitrogen. The flies were homogenized in 0.1 M perchloric acid and then the homogenate was centrifuged at 12 000 g at 4°C for 30 min. Dopamine in the supernatant fluid was measured with an ELISA dopamine kit (LDN, Germany).

#### Cuticular hydrocarbons

To equalize the effects of age, males raised at 31°C were tested when 5-day-old whereas males raised at 25°C were tested when 8-day-old. For hydrocarbon extraction, single flies were immersed for 5 minutes in hexane and removed immediately. The analysis of hydrocarbons was performed following standard procedures [27].

## Supporting Information

Movie S1 Paired-courtship behavior in which wild-type male showed courtship behavior to *ple^ts1^* males reared at 31°C. There is a red color marker on the thorax of *ple^ts1^* male to distinguish *ple^ts1^* male from wild-type male.
(0.23 MB AVI)

## References

[1] Bradbury JW, Vehrencamp SL (1998). Principles of animal communication.

[2] Wyatt TD (2003). Pheromones and Animal Behaviour: Communication by Smell and Taste.

[3] Hall JC (1994). The mating of a fly.. Science.

[4] Yamamoto D, Jallon JM, Komatsu A (1997). Genetic dissection of sexual behavior in *Drosophila melanogaster*.. Annu Rev Ent.

[5] Lasbleiz C, Ferveur JF, Everaerts C (2006). Courtship behavior of *Drosophila melanogaster* revisited.. Animal Behav.

[6] Greenspan RJ, Ferveur JF (2000). Courtship in *Drosophila*.. Annu Rev Genet.

[7] Melis MR, Argiolas A (1995). Dopamine and sexual behavior.. Neurosci Biobehav Rev.

[8] Hull EM, Muschamp JW, Sato S (2004). Dopamine and serotonin: influences on male sexual behavior.. Physiology & Behavior.

[9] Monastirioti M (1999). Biogenic amine systems in the fruit fly *Drosophila melanogaster*.. Microsc Res Tech.

[10] Wright TR (1987). The genetics of biogenic amine metabolism, sclerotization and melanization in *Drosophila melanogaster*.. Adv Genet.

[11] Liu T, Dartevelle L, Yuan CY, Wei HP, Wang Y (2008). Increased dopamine level enhances male-male courtship in *Drosophila*.. J Neurosci.

[12] Neckameyer W (1998a). Dopamine modulates female sexual receptivity in *Drosophila melanogaster*.. J Neurogenet.

[13] Neckameyer W (1998b). Dopamine and mushroom bodies in *Drosophila*: experience-dependent and -independent aspects of sexual behavior.. Learn Mem.

[14] Pendleton RG, Rasheed A, Sardina T, Tully T, Hillman R (2002). Effects of tyrosine hydroxylase mutants on locomotor activity in *Drosophila*: a study in functional genomics.. Behav Genet.

[15] Neckameyer W, Quinn W (1989). Isolation and characterization of the gene for *Drosophila* tyrosine hydroxylase.. Neuron.

[16] Savarit F, Fervuer JF (2002). Temperature affects the ontogeny of sexually dimorphic cuticular hydrocarbons in *Drosophila melanogaster*.. J Exp Biol.

[17] Robertson HM (1983). Mating behaviour and the evolution of *Drosophila mauritiana*.. Evolution.

[18] Eberl DF, Duyk GM, Perrimon N (1997). A genetic screen for mutations that disrupt an auditory response in *Drosophila melanogaster*.. Proc Natl Acad Sci U S A.

[19] Yamamoto D, Nakano Y (1999). Sexual behavior mutants revisited: molecular and cellular basis of *Drosophila* mating.. Cell Mol Life Sci.

[20] Savarit F, Ferveur JF (2002). Temperature affects the ontogeny of sexually dimorphic cuticular hydrocarbons in *Drosophila melanogaster*.. J Exp Biol.

[21] Marican C, Duportets L, Birman S, Jallon JM (2004). Female-specific regulation of cuticular hydrocarbon biosynthesis by dopamine in *Drosophila melanogaster*.. Insect Biochem Mol Biol.

[22] Kopp A, Duncan I, Godt D, Carroll SB (2000). Genetic control and evolution of sexually dimorphic characters.. Nature.

[23] Llopart A, Elwyn S, Coyne JA (2002). Pigmentation and mate choice in *Drosophila*.. Nature.

[24] Villella A, Gailey DA, Berwald B, Ohshima S, Barnes PT (1997). Extended reproductive roles of the *fruitless* gene in *Drosophila melanogaster* revealed by behavioral analysis of new *fru* mutants.. Genetics.

[25] Finley KD, Taylor BJ, Milstein M, McKeown M (1997). *dissatisfaction*, a gene involved in sex-specific behavior and neural development of *Drosophila melanogaster*.. Proc Natl Acad Sci USA.

[26] Sitaraman D, Zars M, LaFerriere H, Chen YC, Sable-Smith A (2008). Serotonin is necessary for place memory in *Drosophila*.. PNAS.

[27] Ferveur JF (1991). Genetic control of pheromones in *Drosophila simulans*. I.*Ngbo*, a locus on the second chromosome.. Genetics.

